# Plasma Metabolomic Profiles Differentiate Patients With Dilated Cardiomyopathy and Ischemic Cardiomyopathy

**DOI:** 10.3389/fcvm.2020.597546

**Published:** 2020-11-10

**Authors:** Junhan Zhao, Shengwen Yang, Ran Jing, Han Jin, Yiran Hu, Jing Wang, Min Gu, Hongxia Niu, Shu Zhang, Liang Chen, Wei Hua

**Affiliations:** ^1^State Key Laboratory of Cardiovascular Disease, Arrhythmia Center, National Center for Cardiovascular Diseases, Fuwai Hospital, Chinese Academy of Medical Sciences and Peking Union Medical College, Beijing, China; ^2^Heart Center & Beijing Key Laboratory of Hypertension, Beijing Chaoyang Hospital, Capital Medical University, Beijing, China; ^3^Peking University First Hospital, Beijing, China; ^4^State Key Laboratory of Cardiovascular Disease, Department of Cardiac Surgery, National Center for Cardiovascular Diseases, Fuwai Hospital, Chinese Academy of Medical Sciences and Peking Union Medical College, Beijing, China

**Keywords:** heart failure, metabolomics, metabolic pathway, dilated cardiomyopathy, ischemic cardiomyopathy

## Abstract

Dilated cardiomyopathy (DCM) and ischemic cardiomyopathy (ICM) are common causes of heart failure (HF). Though they share similar clinical characteristics, their differential effects on cardiovascular metabolomics have yet to be elucidated. In this study, we applied a comprehensive metabolomics platform to plasma samples of HF patients with different etiology (38 patients with DCM and 18 patients with ICM) and 20 healthy controls. Significant differences in metabolomics profiling were shown among two cardiomyopathy groups and healthy controls. Two hundred thirty three dysregulated metabolites were identified between DCM vs. healthy controls, and 204 dysregulated metabolites between ICM patients and healthy controls. They have 140 metabolites in common, with fold-changes in the same direction in both groups. Pathway analysis found the commonalities of HF pathways as well as disease-specific metabolic signatures. In addition, we found that a combination panel of 6 metabolites including 1-pyrroline-2-carboxylate, norvaline, lysophosphatidylinositol (16:0/0:0), phosphatidylglycerol (6:0/8:0), fatty acid esters of hydroxy fatty acid (24:1), and phosphatidylcholine (18:0/18:3) may have the potential to differentiate patients with DCM and ICM.

## Introduction

Heart failure (HF) is a complex and progressive syndrome characterized by the impaired cardiac pump function and inability to meet the metabolic demands of the peripheral tissues, representing a final stage of various cardiovascular diseases (1, 2). HF affects ~1–2% of the adult population worldwide and over 10% of people who are 70 years of age or older and constitutes a major global health burden (3). Despite the rapid development of multiple pharmacologic and device therapies over the past decade, the prognosis of HF patients remains poor, with a mean 5-year survival of 50–60% (4). Current HF management guidelines are mainly based on symptoms and the degree of left ventricular dysfunction, with minimal emphasis on underlying disease etiology, which likely reflects an incomplete understanding of the heterogeneous biological mechanisms contributing to HF (5, 6).

Both dilated cardiomyopathy (DCM) and ischemic cardiomyopathy (ICM) are common causes of HF. Though they share similar clinical characteristics, emerging evidence suggested that they are phenotypically distinct entities and therefore may respond differently to the same therapies (7). Distinguishing between the two diagnoses requires assessment of coronary artery disease (CAD) usually via coronary angiography; however even in the presence of CAD, the diagnosis of DCM should still be considered in patients who have heart failure out of proportion to their degree of CAD (8). Previous studies using RNA sequencing or microarray data have shown that the left ventricles of patients with DCM and ICM exhibited disease-specific gene expression signatures and distinct miRNA networks in the transcription level, providing insight into etiology-specific pathogenesis of HF. This also suggests another mechanism to distinguish between the two diagnoses (9–11).

Metabolites are the most proximal reflection of all the dynamic changes in response to transcriptional and translational regulation as well as posttranslational modifications. Recent advances in metabolomics technology enables comprehensive characterization of low molecular weight metabolites and provided us with an opportunity to investigate the overall snapshot under disease condition (12, 13). To our knowledge, the comparison of metabolic profiling and metabolic dysregulation underlying different subtypes of HF is still lacking.

We applied an untargeted metabolomics profiling to plasma samples of HF patients with different etiology (38 patients with DCM and 18 patients with ICM, respectively), and 20 healthy controls, and comparatively assessed the metabolomics profiles in different cardiomyopathies. By using a comprehensive untargeted metabolomics strategy, we aimed to explore the systemic metabolomics signatures and dysregulation pathways to resolve distinct etiologies within HF, and to develop a biomarker panel that may be beneficial for differential diagnosis.

## Materials and Methods

### Study Population

HF patients were consecutively enrolled in Fuwai hospital in Beijing China between June 2018 and July 2019. The HF diagnosis was made according to the recent guidelines (14). Patients with DCM were eligible to enroll after their diagnosis was confirmed, as defined by left ventricular or biventricular systolic dysfunction and dilatation that are not explained by abnormal loading conditions or coronary artery disease (15). ICM patients were diagnosed with history of MI or revascularization (CABG or PCI) or patients with more than 75% stenosis of left main or proximal LAD or patients with more than 75% stenosis of two or more epicardial vessels (8). The control group was composed of 20 healthy individuals. Healthy controls were recruited from normal non-blood related relatives of patients with heart failure (except for some mutation carriers of hereditary cardiomyopathy). And they all underwent echocardiography and electrocardiogram examination to exclude heart disease. The clinical characteristics of all participants were obtained from the electronic health records. Exclusion criteria were: (1) Age <18; (2) Patients with systemic complications such as malignant tumor, autoimmune disease, endocrine disease, or end-stage renal dysfunction; (3) blood-borne infectious diseases, including human immunodeficiency virus/acquired immunodeficiency syndrome, hepatitis B, and hepatitis C; (4) Absence of blood samples. Finally, 38 patients with DCM and 18 patients with ICM were selected for further study. All individuals signed informed consent. This investigation conformed to the principles outlined in the Declaration of Helsinki and was approved by institutional ethics board of Fuwai hospital.

### Sample Collection and Preparation

All participants were informed to avoid strenuous exercise and alcohol consumption within 24 h before blood collection. Peripheral blood samples were drawn from cubital vein in fasting state in the morning before 9 a.m. We collected blood samples in EDTA VACUETTE tube (Greiner Bio-one, Thailand) and gently shook the tubes 4–5 times to mix up blood sample with anticoagulant. Then the tubes were centrifuged at 4,000 r/min for 10 min at 4°C. Plasma was immediately separated and stored at −80°C refrigerator. Samples were thawed on ice before preparation. Four hundred micro liter of cold methanol (containing internal standard) was added to 100 μL of plasma sample and vortexed for 60 s. After 10 min at room temperature, they were centrifuged at 14,000 g for 15 min to precipitate the protein. And the supernatant was then transferred and collected for the analysis in positive electrospray ionization (ESI+) mode and negative electrospray ionization (ESI-) mode, respectively. To evaluate the stability of the analysis, quality control (QC) samples were prepared by mixing equal volumes of each plasma sample and evenly injected at regular intervals throughout the analytical run.

### Metabolomics Analysis

A Dionex Ultimate 3000 RS UHPLC system (Thermo Fisher Scientific, Waltham, MA, USA) was equipped with ACQUITY UPLC BEH C18 column (1.7 μm, 2.1 × 100 mm, Waters Corp, Milford, United States) for ESI+ mode and UPLC HSS T3 column (2.1 mm × 100 mm, 1.8 μm, Waters Corp, Milford, United Sates) for ESI- mode. In ESI+ mode analysis, the binary gradient elution system consisted of (A) water (containing 0.1% formic acid, v/v) (B) acetonitrile (containing 0.1% formic acid, v/ v). The separation was accomplished through the following gradient: B start at 5%, 1–24 min to reach 100%, the composition was held at 100% B, then 27.5–27.6 min, 100 to 5% B, and 27.6–30 min holding at 5% B. In ESI- mode analysis, the mobile phase A was 6.5 mM ammonium acetate in water and mobile phase B was 6.5 mM ammonium acetate in 95% methanol. The separation was accomplished through the following gradient: B start at 5%, 1–18 min to reach 100%, the composition was held at 100% B 18.1–22 min, then 22–22.1 min, 100 to 5% B, and 22.1–25 min holding at 5% B. The flow rate and column temperature were same as ESI+ mode.

The injection volume was 5 μl. Liquid chromatograph tandem mass spectrometer (LC–MS/MS) analysis was performed on a Q-Exactive quadrupole-Orbitrap mass spectrometer (Thermo Fisher Scientfic, Waltham, MA, United States) equipped with heated electrospray ionization (ESI) source in both ESI+ and ESI- modes. Data acquisition was performed in full scan mode combined with Data Dependent Acquisition (DDA) mode. The mass spectrometer parameters were set as following: spray voltage, 3800V (ESI+) and 3000V (ESI-); capillary temperature, 320°C; Aux gas heater temperature: 350°C; sheath gas flow rate, 35 arbitrary units; auxiliary gas flow rate, 8 arbitrary units; S-lens RF level: 50. The mass range was from m/z 70 to 1,000. The resolution was set at 70,000 for the full MS scans and 17,500 for MS/MS scans. The Collision energy was set at 20, and 40eV in NCE mode.

### Data Processing and Metabolite Identification

Metabolomics data were acquired using the XCMS software (1.50.1). The preprocessing procedure generated a data matrix that consisted of the retention time, mass-to-charge ratio (m/z) values, and peak intensity. All ions were normalized to the total peak area of each sample. If a variable had a non-zero measurement value in at least 85% of the variables within one of the two subsets, the variable was included in the data set; otherwise the variable was removed. OSI-SMMS (version 1.0, Dalian Chem Data Solution Information Technology Co. Ltd.) was used for peak annotation after XCMS data processing with an in-house MS/MS database. Data has been deposited to the EMBL-EBI MetaboLights database (MTBLS2155) (16).

### Statistical Analysis

Clinical characteristics were exhibited as the mean ± SD for continuous variables and as the number (percent) for categorical variables. Continuous variables of two groups were compared using the Student's *t*-test (for normally distributed) and Wilcoxon test (for non-normally distributed). Categorical variables were compared by the Chi-squared test or Fisher's exact test as appropriate. Metabolomics data were z-score normalized before analysis. Principle component analysis (PCA) and partial least squares discriminant analysis (PLS-DA) were used to discriminate among different groups in unsupervised and supervised mode, respectively. R2Y (cum), Q2 (cum), and permutation test were used to evaluate the stability and predictability of the model. Differential metabolites by disease groups (DCM vs. healthy controls and ICM vs. healthy controls) adjusted for age, gender, and medication use were determined by linear modeling using the lmFit and eBayes functions implemented in the limma R package (17, 18). The *P*-values were adjusted by Benjamini–Hochberg method to account for multiple testing. The pathway analysis with the topology analysis was used to identify the most relevant metabolic pathways. The least absolute shrinkage and selection operator (LASSO) implemented in glmnet R package was applied to determine non-zero coefficient features to identify the most important metabolites in model prediction. The tuning parameter (λ) was selected used 10-fold cross-validation via minimum criteria and the 1 standard error (1-SE). The model that minimized CV error plus one standard error was chosen as the final model. The regression model was established by a multivariable logistic regression. The performance of prediction accuracy assessment was conducted by a receiver operating characteristic (ROC) curve in pROC R package. To evaluate the performance of the classification model, the dataset was randomly divided into training set and testing set and cross validation was performed 500 times. The statistical analysis was performed by R x64 3.6.2, SPSS Statistics 22.0 (IBM, New York, United States), SIMCA-P software 14.1, (Umetrics, Umea, Sweden), and MetaboAnalyst 4.0 online website (19).

## Results

### Baseline Characteristics of Patients With DCM, Patients With ICM and Healthy Controls

**Figure 1:**
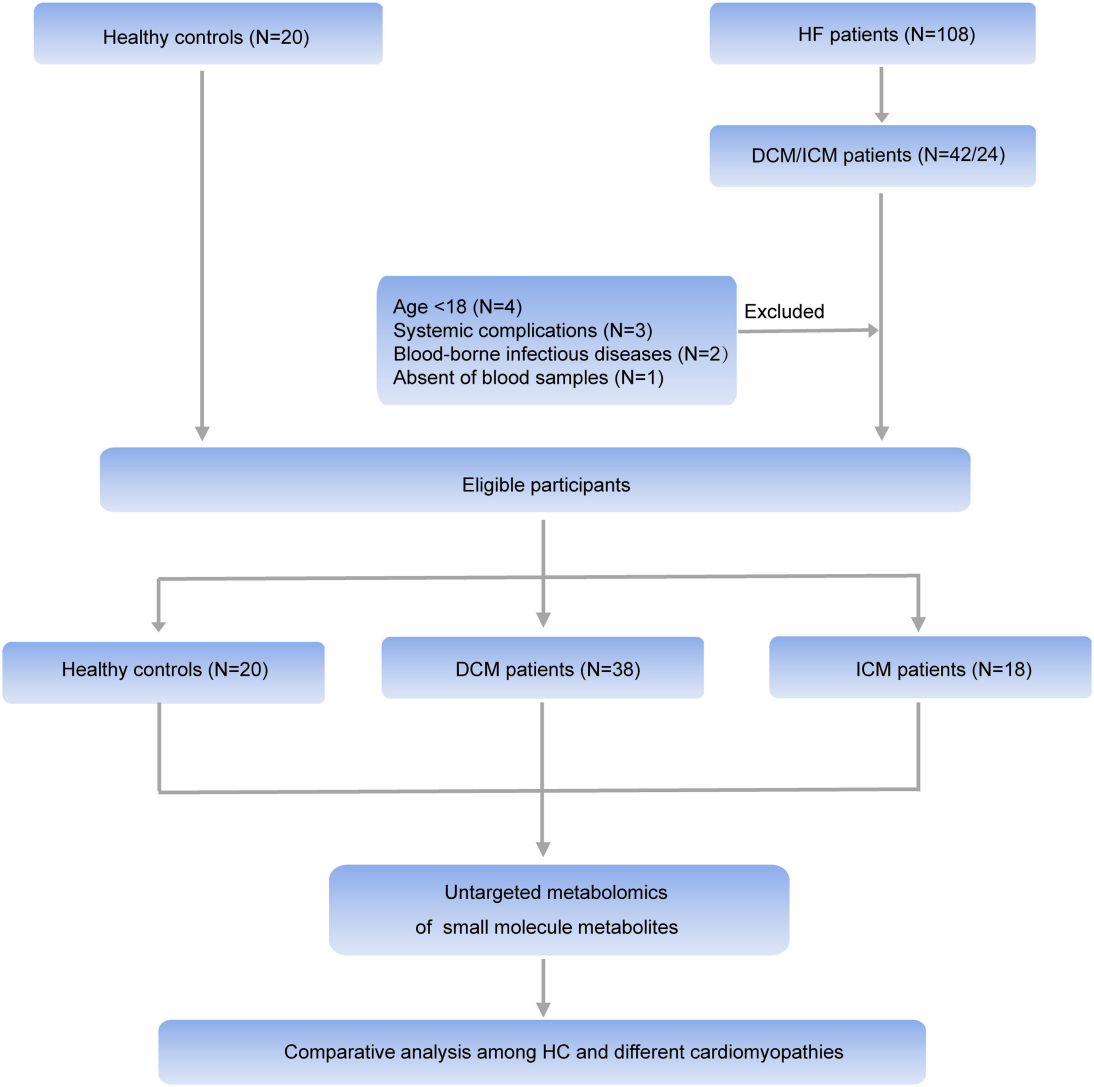
Workflow of the present study. In general, HF patients with different etiology (38 patients with DCM and 18 patients with ICM, respectively), and 20 healthy controls were enrolled in this study. An untargeted metabolomic panel was applied to the plasma samples of these individuals. We comparatively analyzed the plasma metabolomics profiling of the 3 groups to clarify the differences in HF patients with different etiology from the perspective of circulation metabolomics.

Comprehensive untargeted metabolomics technique was employed to assess the plasma samples. Briefly, 108 HF patients were consecutively enrolled in Fuwai hospital and patients with the diagnosis of DCM or ICM were selected in this study. Ten patients were excluded according to the exclusion criteria. Thirty eight patients with DCM, 18 patients with ICM were finally selected for further study (Figure 1). Their clinical characteristics was displayed in Table 1. Age, gender and BMI are comparable among healthy controls, patients with DCM and patients with ICM. And no significant differences in HF related biomarkers such as CK-MB, NT-proBNP, and hs-CRP were shown between patients with DCM and patients with ICM, indicating comparable HF severity. The incidences of hyperlipidemia were lower in patients with DCM than patients with ICM (42.1 vs. 100%, *P* < 0.0001). And all the patients received routine medication therapy for HF, and their drug administering for statin (38.1 vs. 100%, *P* < 0.0001 and aspirin (11.9 vs. 59.1%, *P* = 0.0004) were different between these two groups.

**Table 1:** Clinical characteristics of DCM and ICM patients.

**Variable**	**Healthy controls** ***N* = 20**	**DCM patients** ***N* = 38**	**ICM patients** ***N* = 18**	***P*-value**
**Baseline**				
Age, yrs	55.2 ± 9.5	57.2 ± 10.3	60.0 ± 8.7	0.313
Gender, male	12 (60.0%)	23 (60.5%)	12 (66.6%)	0.890
BMI, kg/m^2^	25.3 ± 2.6	25.6 ± 3.1	24.8 ± 3.5	0.601
SBP, mmHg		121.6 ± 15.0	118.4 ± 15.8	0.472
DBP, mmHg		70.7 ± 7.5	69.6 ± 10.4	0.631
HR, beats/min		69.4 ± 10.1	69.4 ± 11.6	0.980
NYHA functional classI/II/III/ IV		0/12/16/10	0/6/7/5	0.974
**Echocardiography**				
LVEDD, mm	45.7 ± 3.8	66.3 ± 9.9	62.6 ± 8.8	0.186
LVEF, %	64.9 ± 9.1	35.8 ± 12.4	37.0 ± 11.4	0.731
Lad, mm	33.3 ± 3.2	35.8 ± 12.4	40.1 ± 4.0	0.335
**Serum biomarkers**				
Scr, umol/L		87.5 ± 16.3	88.0 ± 14.6	0.917
Hs-CRP, mg/L		2.3 ± 2.5	2.5 ± 3.0	0.842
NT-proBNP, pg/ml		1,267.0 ± 2,300.2	1,466.1 ± 1,299.6	0.701
Uric acid, umol/L		396.1 ± 126.8	387.4 ± 148.2	0.826
Albumin, g/L		43.3 ± 5.1	42.9 ± 5.0	0.799
CK-MB, U/L		12.2 ± 4.3	17.6 ± 22.6	0.159
**Comorbidity**				
Hypertension		14 (36.8%)	8 (44.4%)	0.360
Diabetes		14 (36.8%)	9 (50.0%)	0.394
Atrial fibrillation		3 (7.8%)	3 (16.7%)	0.389
Hyperlipidemia		16 (42.1%)	18 (100%)	<0.0001
**Medication**				
ACEI/ARBs		36 (94.7%)	14 (77.8%)	0.077
Beta-blockers		35 (92.1%)	17 (94.4%)	0.999
Spironolactone		35 (92.1%)	14 (77.8%)	0.195
Trimetazidine		10 (26.3%)	7 (38.9%)	0.366
Statin		14 (36.8%)	18 (100.0%)	<0.0001
Aspirin		5 (13.2%)	11 (61.1%)	0.0004

*BMI, body mass index; SBP, systolic blood pressure; DBP, diastolic blood pressure; HR, heart rate; NYHA, New York Heart Association; LVEDD, left ventricular end diastolic diameter; LVEF, left ventricular ejection fraction; Lad, left atrial diameter; Scr, Serum creatinine; Hs-CRP, high-sensitivity C-reactive protein; NT-proBNP, N-terminal pro-B-type natriuretic peptide; CK-MB, creatine kinase MB; ACEI/ARBs, angiotensin-converting enzyme inhibitor/angiotensin receptor blockers. Data are expressed as the mean ± standard deviation (SD) or as number (%)*.

### Metabolomics Profiling Among Patients With DCM, ICM and Healthy Controls

Four hundred forty nine endogenous metabolites were acquired in ESI+ mode, and 338 endogenous metabolites were acquired in ESI- mode. 75.57 and 72.62% of features in QC samples in ESI+ mode and in ESI- ion mode possessed a relative standard deviation (RSD) <30%, which displayed good reproducibility and stability of the metabolomics method (Supplementary Figure 1). QC samples in PCA score plot (Supplementary Figure 2) clustered together, indicating good reproducibility of the sample processing and the instrumental system.

**Figure 2:**
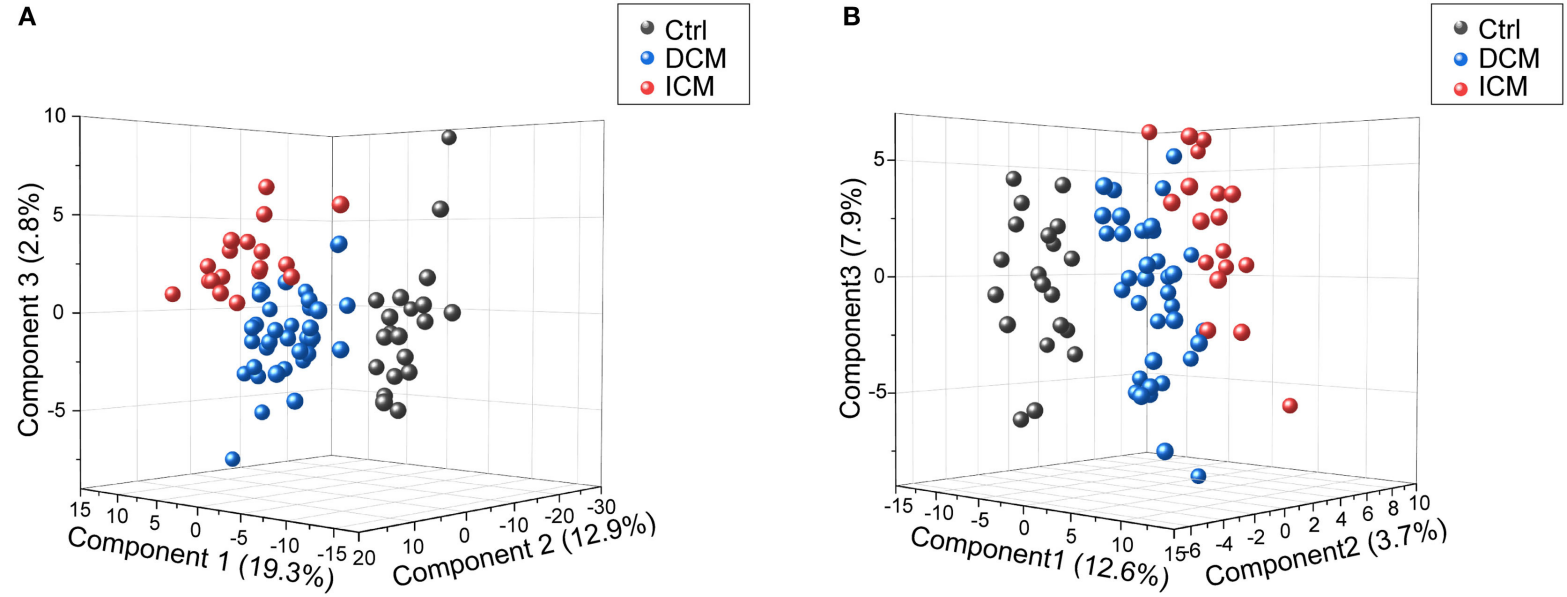
Score plots of PLSDA model in positive and negative ion modes. In the three-dimensional score plots, each plot represents a sample. They showed a distinct classification in healthy controls (gray) and patients with DCM (blue) and ICM (red) in positive ion mode **(A)** and negative ion mode **(B)**.

In order to comparatively analyze the metabolomics profiling among three groups, PCA and PLS-DA was applied to visualize the differences between DCM and ICM in both ESI+ and ESI– mode (Supplementary Figure 3, Figure 2). As shown in score plots in Figure 2, there is a distinct classification for each group of individuals in both ESI+ mode (Figure 2A) and ESI- mode (Figure 2B), indicating significant different metabolomic signatures among three groups. Evaluation of the PLS-DA model with their corresponding R2Y and Q2 and permutations test suggested a good stability and predictability of these two models (Supplementary Figures 4, 5). The plasma metabolomics of HF patients is quite different from that of healthy controls. In addition, the HF patients caused by different diseases also clustered into two groups, suggesting a profound metabolic alteration between DCM and ICM patients.

### Differential Metabolites Between Patients With DCM, ICM and Healthy Controls

**Figure 3:**
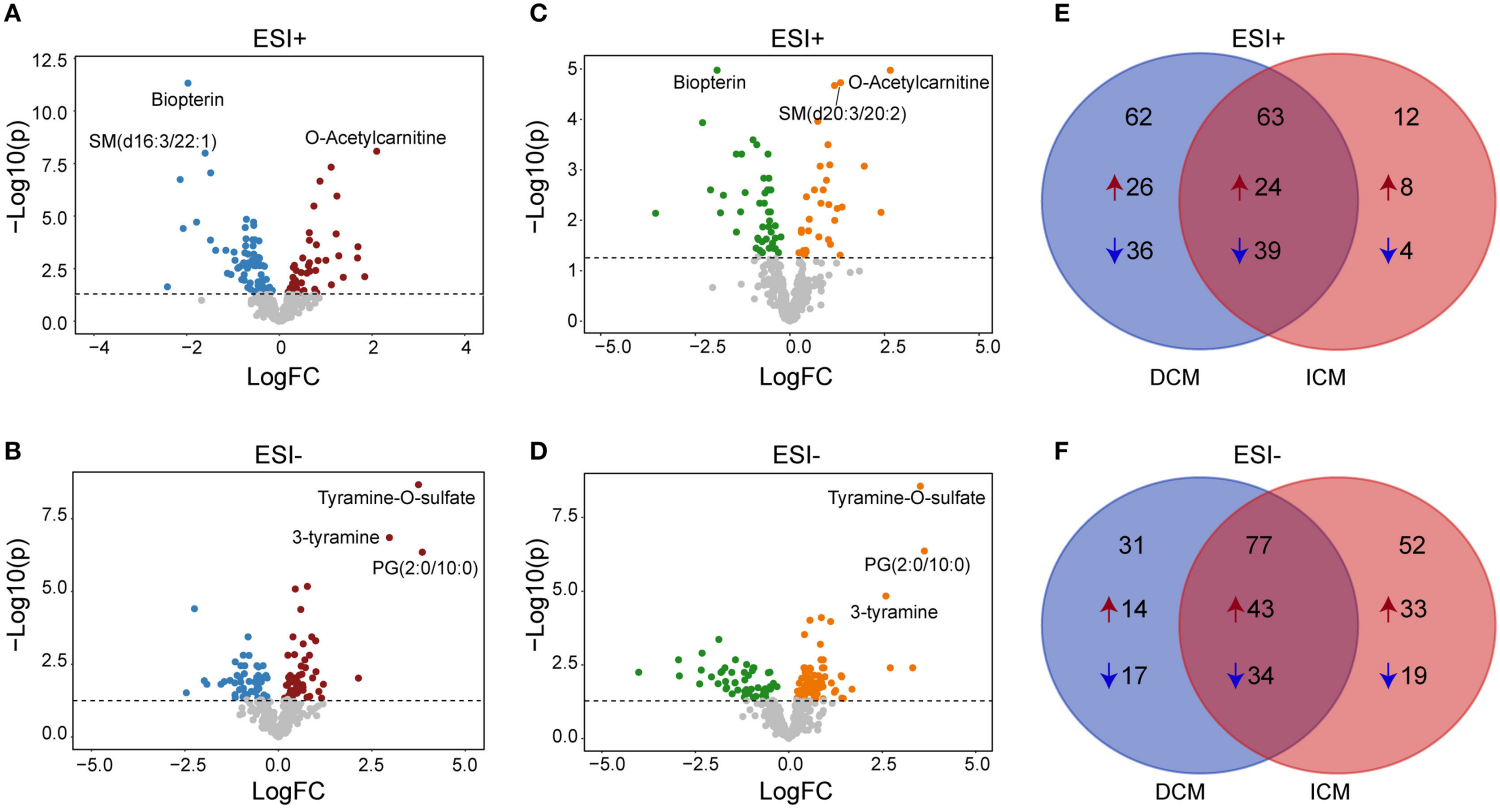
Differential metabolites between patients with DCM, ICM, and healthy controls. Volcano plots for dysregulated endogenous metabolites between patients with DCM and healthy controls in positive ion mode **(A)** and negative ion mode **(B)**. Volcano plots for dysregulated endogenous metabolites between patients with ICM and healthy controls in positive ion mode **(C)** and negative ion mode **(D)**. Each plot represents a metabolite. The differentiating metabolites with adjusted *P* < 0.05 were highlighted in color. The venn diagram displayed the differential metabolites shared with DCM and ICM and the specific metabolites to be significant only in one comparison in in positive ion mode **(E)** and negative ion mode **(F)**.

We next performed a differential expression analysis adjusted for age, gender and medication use, to identify metabolites dysregulated between DCM vs. healthy controls, ICM vs. healthy controls and also to reveal a core set of dysregulated metabolites common to both cardiomyopathies. In DCM vs. healthy controls, 125 significant differential endogenous metabolites (Benjamini–Hochberg adjusted *P* < 0.05) were identified with 50 up-regulated and 75 down-regulated metabolites in ESI+ mode (Figure 3A). One hundred eight differential metabolites (Benjamini–Hochberg adjusted *P* < 0.05) were identified with 57 up-regulated and 51 down-regulated metabolites in ESI- mode (Figure 3B). Meanwhile, in the comparison between ICM and healthy controls, we identified 75 differential metabolites (Benjamini–Hochberg adjusted *P* < 0.05) with 32 up-regulated and 43 down-regulated metabolites in ESI+ mode (Figure 3C), And 129 differential metabolites (Benjamini–Hochberg adjusted *P* < 0.05) with 76 up-regulated and 53 down-regulated metabolites in ESI-mode (Figure 3D).

The venn diagram displayed the differential metabolites shared with DCM and ICM and the specific metabolites to be significant only in one comparison (Figures 3E,F, Supplementary Tables 1–3). The number of common differential metabolites shared with DCM and ICM were 63 in ESI+ mode (Figure 3E) and 77 in ESI- mode (Figure 3F), respectively. Totally, 40 metabolites were elevated and 53 metabolites were reduced only in patients with DCM with no significant changes in patients with ICM. Forty one metabolites were elevated and 23 metabolites were reduced only in patients with ICM with no significant changes in patients with DCM.

### Heart Failure Common Differential Metabolites Shared With DCM and ICM Patients

In the two comparisons of DCM vs. healthy controls and ICM vs. healthy controls, they have 140 metabolites in common, with fold-changes in the same direction, indicating that these metabolites were related to HF common changes (Supplementary Table 1). The heatmap consisting of the shared metabolites (fold change>2 or <0.5, Benjamini–Hochberg adjusted *P* < 0.05) was shown in Supplementary Figure 6. O-Acetylcarnitine, as an indication of carnitine level and acetyl-CoA availability, was found to be increased in both DCM (fold change = 4.2, *P* = 8.20E-09) and ICM (fold change = 6.2, *P* = 1.06E-05). Tyramine-O-sulfate and 3-tyramine, as derivative of tyrosine, phenylethylamine and other catecholamines, were both increased in DCM (fold change = 13.4, *P* = 2.16E-19; fold change = 7.8, *P*= 1.42E-16) and ICM patients (fold change = 11.5, *P*= 2.74E-09; fold change = 6.0, *P* = 1.46E-05). Meanwhile, consistent with previous studies in HF, we also found decreased level of amino acids like ornithine and citrulline, tricarboxylic acid cycle (TCA) intermediates alpha-ketoglutaric acid, and steroid hormones dehydroepiandrosterone sulfate and 5α-pregnane-3,20-dione in DCM and ICM (20–22).

### Dysregulated Pathway Observed in DCM vs. ICM

**Figure 4:**
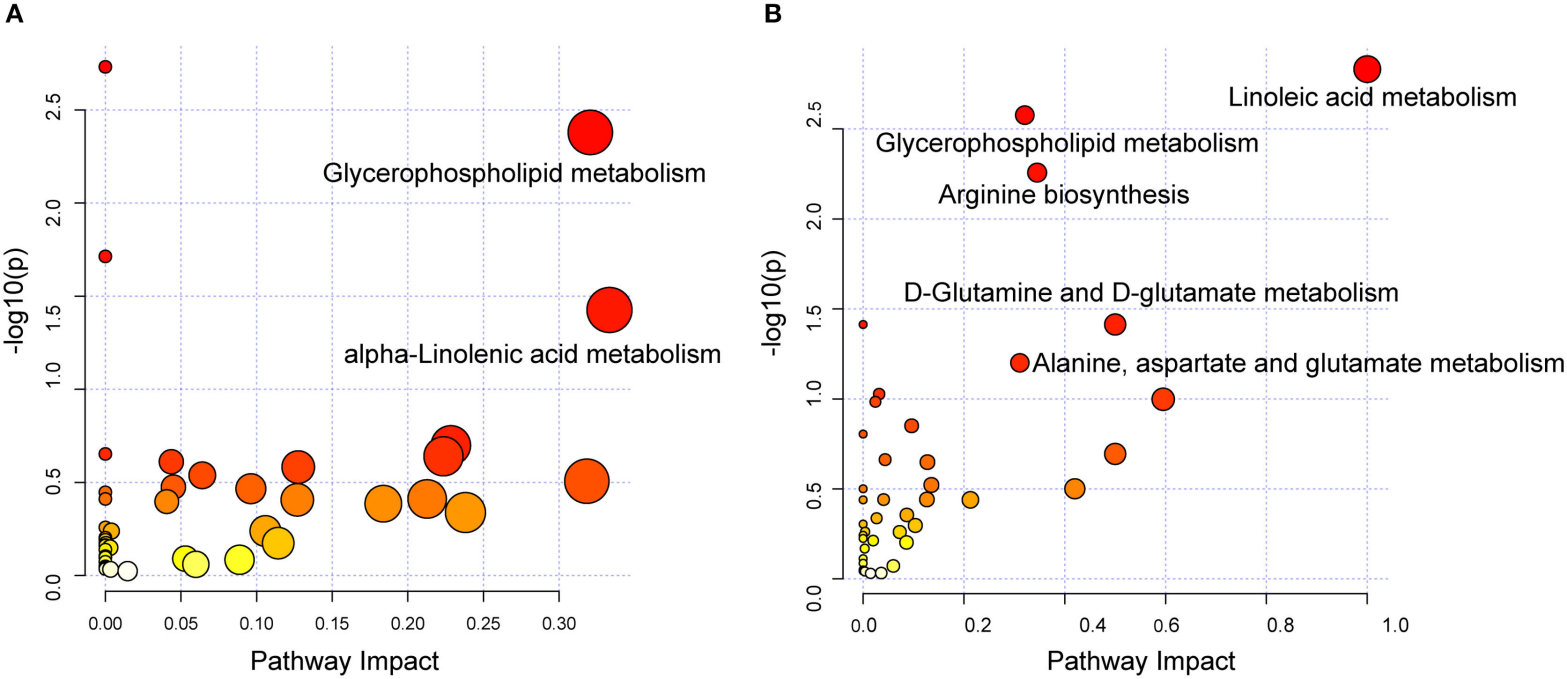
Dysregulated pathway observed in DCM and ICM. Dysregulated pathways involved in the pathogenesis of patients with DCM **(A)** or ICM **(B)**. Significant pathways (*P* < 0.05) were highlighted.

To investigate the biological pathways involved in the metabolic signatures of DCM and ICM, pathways analysis was conducted using the significantly changed metabolites. The pathway analysis results were shown in Figures 4A,B and Supplementary Tables 4, 5. Glycerophospholipid metabolism were identified to be significant in both DCM and ICM group. And alpha-linolenic acid metabolism was identified as significant pathway in DCM group and linoleic acid metabolism and arginine biosynthesis, D-Glutamine and D-glutamate metabolism were identified as significant pathway in ICM group.

### Disease Specific Metabolites Differentiate Patients With DCM and ICM

**Figure 5:**
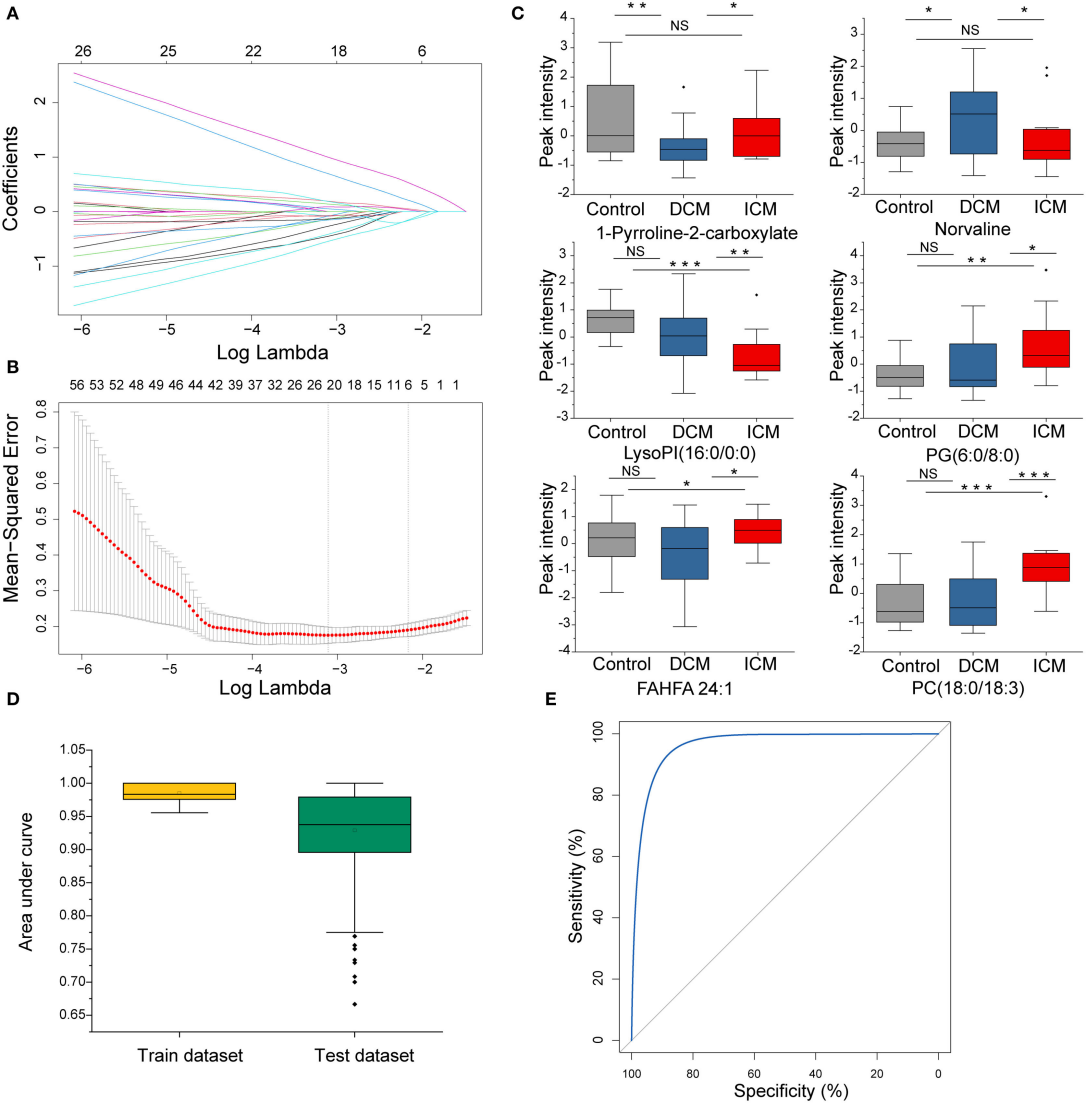
The disease specific dysregulated metabolites distinguish patients with DCM from patients with ICM. **(A)** LASSO coefficient profiles of the disease-specific features. A coefficient profile plot was produced against the log(λ) sequence. Dotted vertical line was drawn at the optimal λ at minimum criteria and 1 standard error (1-SE criteria). The model at 1-SE criteria was selected as the final model with 6 non-zero coefficients. **(B)** Tuning parameter (λ) selection in the LASSO model used 10-fold cross-validation via minimum criteria. The binomial deviance was plotted vs. log (λ). **(C)** Bar plot of the 6 metabolites in DCM and ICM and HC. Student's *t*-test for two group comparison. **(D)** Plot of the area under curve for the created biomarker model in training and testing dataset by cross validation under 500 random replications. Data are presented as mean ± SD in bar plots. **(E)** The ROC created by a combination of six selected features based on whole dataset. *P < 0.05, **P < 0.01, ***P < 0.001.

In order to select the most significant metabolites that distinguish patients with DCM from patients with ICM, LASSO logistic regression model was applied to the disease specific dysregulated metabolites. The optimal λ values at 1 standard error (1-SE criteria) with six metabolites were selected (Figures 5A,B). These six metabolites were found to be significantly different between patients with DCM and patients with ICM, including phosphatidylcholine 1-pyrroline-2-carboxylate, norvaline, lysophosphatidylinositol(16:0/0:0), phosphatidylglycerol(6:0/8:0), fatty acid esters of hydroxy fatty acid (24:1) and phosphatidylcholine(18:0/18:3) (Figure 5C). They remained significant after adjusting for confounders including hyperlipidemia and drug use of ACEI, statins and aspirin. The regression model was established by a multivariable logistic regression based on a panel of the six metabolites. The dataset was randomly divided into training set and testing set for 500 times. The median AUCs of the ROC were 0.98 and 0.93 with accuracy of 95% and 87% in training and testing dataset, respectively, indicating an excellent discrimination of the combination model for these panel (Figure 5D). The ROC curve based on the whole dataset possess areas under the curve of 0.97 (95% CI, 0.94–1.0) (Figure 5E). These six metabolites may have the potential to suggest different etiologies in HF.

## Discussion

In this study, plasma metabolomics of HF patients with different etiology (patients with DCM and ICM) and healthy controls were assessed through comprehensive metabolomics technology. We found that both DCM and ICM patients are distinct from healthy controls in metabolomics profiling, and they share some common dysregulated metabolites and metabolic pathways. Furthermore, we also found that DCM and ICM exhibited different metabolomics profiling and the identified pathways that specialized in each disease, highlighting the role of metabolic dysregulation related to different disease mechanism. Lastly, we created a panel of six metabolites that could distinguish DCM from ICM, which may have the potential to serve as a tool to suggest different etiology in HF.

### Heart Failure Common Differential Metabolites

HF is a complex syndrome which is accompanied by a number of metabolic perturbations (23). The significantly different metabolic profiling between HF and healthy controls indicated systemic metabolic alteration in the disease. Impaired fatty acid beta oxidation, TCA cycle, glycerophospholipid metabolism and arginine biosynthesis has been revealed in our study as well as previous findings (20, 24, 25). Branched chain amino acid (BCAA) metabolism was not identified, however, as BCAAs and their metabolites were not detected by this approach.

### Metabolites Biomarkers Suggest Different Etiology in HF

The findings of the present study demonstrate that plasma metabolites biomarkers can identify etiology in HF. DCM and ICM are indeed distinct diseases and they possessed different survival and respond differently to therapies (26, 27). Their differential diagnosis is highly dependent on invasive assessment like coronary artery angiography. And it is still challenging for differential diagnosis, especially in patients with heart failure out of proportion to their coronary artery disease, up to 11% in one observational study (8). This plasma profile that distinguishes DCM and ICM could provide a valuable adjunct to imaging tools and serve as classification biomarkers in future application in differential diagnosis.

1-Pyrroline-2-carboxylate, a product of proline, is involved in the D-arginine and D-ornithine metabolism pathway and the arginine and proline metabolism pathway. Proline and its metabolites are involved in redox balance and act as reactive oxygen species scavenger (28, 29). The downregulation of 1-pyrroline-2-carboxylate in plasma of DCM patients may implicate decreased capacity against oxidative stress. Norvaline, an unbranched-chain amino acid, it is known to promote NO production through inhibiting arginase and also exerts anti-inflammatory effects on endothelium through a mechanism due to inhibition of the TNFα-induced S6K1 activation, leading to a decrease in the expression of adhesion molecules (30, 31). However, human studies on plasma norvaline are lacking and further studies related to DCM are necessary. Cellular lipids are the major component of cell membranes and play a crucial role for both cellular and physiological energy homeostasis (32). Phosphatidylglycerol (6:0/8:0), fatty acid esters of hydroxy fatty acid (24:1) and phosphatidylcholine (18:0/18:3) were all elevated, while lysophosphatidylinositol (16:0/0:0) were decreased in patients with ICM rather than DCM when compared to healthy controls. Lysophosphatidylinositol (lysoPI) is a bioactive lipid generated by phospholipase A (PLA). LysoPI and its receptor G-protein coupled receptor 55 (GPR55) has been implicated to have a potential role in the pathogenesis of HF by controlling the adrenergic signaling pathway in the heart (33). Also lysoPI levels could be regulated by cellular conditions associated with ischemia and reperfusion and have possible important implications for improving atherosclerosis (34). Phosphatidylglycerols (PG) is a precursor for cardiolipin synthesis, who presented in inner mitochondria membrane of and is vita in maintaining the structural and functional integrity of mitochondrial respiratory complexes (35). Significantly increased cardiolipin has also been implicated in ischemic cardiomyopathy model (36). Fatty acid esters of hydroxy fatty acids (FAHFAs) are recently discovered endogenous lipids with antidiabetic and anti-inflammatory properties. It is found in adipose tissue and serum that correlate with insulin sensitivity and are reduced in insulin-resistant humans (37, 38). The biosynthetic pathway and origin of FAHFAs is still not clear and also their role regarding to cardiovascular disease remained to be discovered. Dyslipidemia are seen in most patients with ICM. Phosphatidylcholine (PC) is the predominant phospholipid component of circulating lipoproteins (39). Increased plasma PC level were associated with cardiovascular disease in a prospective study (40). And inhibition PCs biosynthesis reduces atherosclerosis and prevents cardiac dysfunction (39).

### Etiology-Specific Mechanisms of HF

The disease-specific metabolic pathways may give insight into etiology-specific pathogenesis of HF and thus pave the way for precision therapy in HF management. Despite the historical view of atherosclerosis as a lipid storage disease, the involvement of pro-inflammatory monocytes and local tissue macrophages as major protagonists in the generation and progression of atherosclerotic lesions has expanded our understanding of its pathophysiology (41, 42). Linoleic acid metabolism including an increase in n-6 polyunsaturated fatty acids, arachidonic acid as well as its metabolites has been emphasized in ICM patients. Cyclooxygenase -derived eicosanoids deriving from arachidonic acid were increased in ICM and they contribute to inflammatory responses associated with myocardial dysfunction as well as the progression of atherosclerotic lesions (42, 43). These findings further highlight the COX pathway as vital treatment targets in ICM patients. In addition, the impaired energy utilization in ICM resulted in increased glutaminolysis, which can produce α-ketoglutarate to replenish the Krebs cycle, serving as an anaplerotic source of carbon for the formation of non-essential amino acids and lipids (44, 45). In addition, glutamate is used for the synthesis of the antioxidant glutathione, which promotes redox and limiting oxidative stress through maintenance of cardiac glutathione metabolism (46). Glutamine supplementation protects against cardiac injury and restore cardiac function (47, 48). The therapeutic strategy regarding glutamine and glutamate metabolism may be considered in future personalized treatment for ICM. Furthermore, Arginine biosynthesis, involved in a number of biological processes, including the biosynthesis of proteins, host immune response, urea cycle, and nitric oxide production, plays an important role in the regulation of endothelial function and vascular tone, which is vital for ICM treatment (23, 49). alpha-linolenic acid metabolism was observed in both DCM and ICM, but it found to be significant only in DCM group. A relationship between increased intake of n-3 polyunsaturated fatty acids and beneficial effects on the cardiovascular system has become acknowledged throughout the years. n-3 PUFAs exhibit positive effects on hemostatic factors, thrombogenesis, blood pressure, plasma lipids, and heart susceptibility to ventricular arrhythmias (50, 51).

Gender differences can influence every facet of HF, from epidemiology and risk factors, to pathophysiology and prognosis (52). It has been showed a different gene expression pattern in female hearts with up-regulation of the genes regarding to energetic metabolism and down-regulation of genes regarding to fibrosis and inflammation, indicating a better response to adverse stimuli (53–55). Recent studies have suggested gender-specific perturbations of some metabolites like microbiome-derived metabolite trimethylamine N-oxide (TMAO) in heart tissues. TMAO was elevated in male ICM and DCM compared to male donor hearts, whereas it was not significantly different between female HF and female donor hearts (18). These findings permitted a better understanding of divergent HF pathogenesis between males and females.

## Limitations

Several limitations should be mentioned for the present study. First, we used only plasma samples instead of heart tissues samples, the latter of which are much more difficult to obtain. The plasma metabolomics changes can reflect the contribution of several organs. We cannot determine the underlying causes leading to these alterations in plasma metabolites. Thus, the identified metabolic changes cannot reflect changes in cardiac metabolism. Second, our study shed light on the metabolic dysregulation in HF; however, more detailed investigation about their role of these metabolites in the pathogenesis remains to be elucidated in future preclinical studies. Third, the identified biomarker panel, in spite of its good performance, was explored in a single center with relatively small sample size. Multi-center studies with larger populations as external validation cohort is needed in our future studies.

## Conclusion

In this study, a comprehensive metabolomics analysis of HF patients revealed significant different plasma metabolomics signatures with different cardiomyopathy. And we developed a biomarker panel that may have the potential to differentiate patients with DCM and ICM. The commonalities as well as disease-specific metabolic signatures in DCM and ICM could provide more insights into disease underlying mechanisms as well as personalized therapy strategy. External validation and preclinical studies regarding to the dysregulated metabolism underlying different cardiomyopathy are needed in future studies.

## Data Availability Statement

The datasets presented in this study can be found in online repositories. The names of the repository/repositories and accession number(s) can be found below: EBI MetaboLights database [accession: MTBLS2155].

## Ethics Statement

The studies involving human participants were reviewed and approved by Institutional Ethics Board of Fuwai Hospital. The patients/participants provided their written informed consent to participate in this study.

## Author Contributions

WH and LC contributed to the study conception and design. Material preparation and data collection were performed by RJ, YH, HJ, JW, and MG. JZ and SY analyzed the data. The first draft of the manuscript was written by JZ and all authors commented on previous versions of the manuscript. All authors read and approved the final manuscript.

## Conflict of Interest

The authors declare that the research was conducted in the absence of any commercial or financial relationships that could be construed as a potential conflict of interest.
