# Supplementary material for: Plasma Metabolomic Profiles Differentiate Patients With Dilated Cardiomyopathy and Ischemic Cardiomyopathy
Source: Front Cardiovasc Med. 2020 Nov 10;7:597546. doi: 10.3389/fcvm.2020.597546 (PMC7683512; doi:10.3389/fcvm.2020.597546)
Supplement: Supplementary file 1 [file Data_Sheet_1.docx]

Supplementary Material

# Supplementary figures

A B


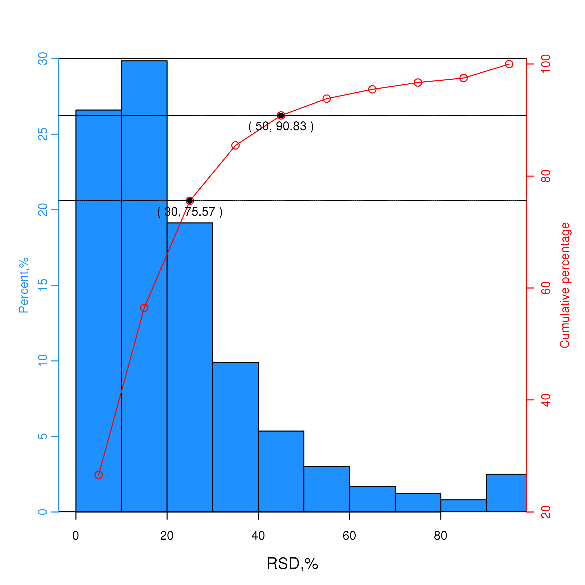

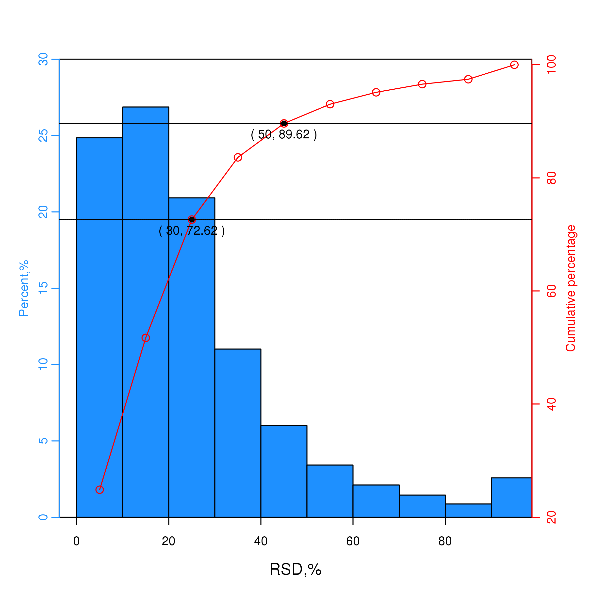


## Supplement figure 1 Relative standard deviation of mass spectra in QC samples.

The X axis is the relative standard deviation (RSD) of the mass spectrometry features, and the Y axis is the percentage of the features with RSD in this range. The curve in the figure is the cumulative value of the percentages in different RSD intervals. Figure 1A represented the condition in positive ion mode and figure 1B for negative ion mode.

A B

##
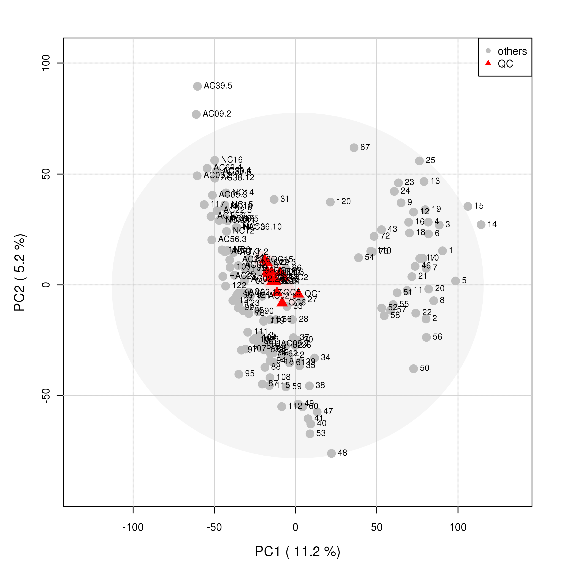

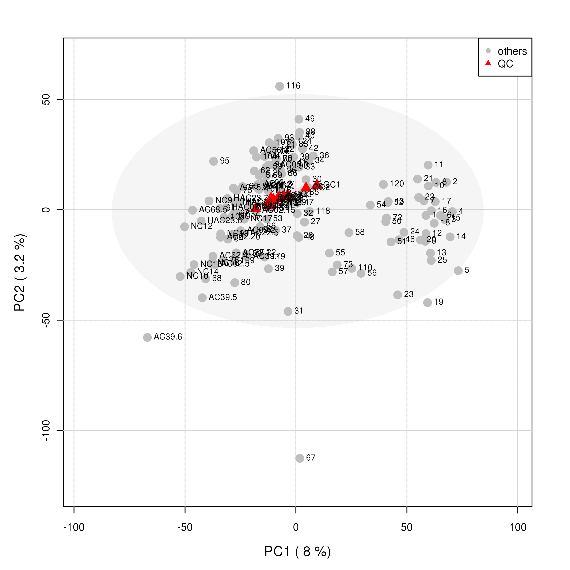


## Supplement figure 2 PCA plot of QC samples.

## The X axis is PC1, and the Y axis is PC2. The red triangles in the PCA plot represent QC samples, and the grey dispersion points represent all the remaining samples. Figure 2A represented the condition in positive ion mode and figure 2B for negative ion mode.

A B







## Supplement figure 3 PCA plot in positive and negative ion modes.

## The X axis is PC1, and the Y axis is PC2 and Z axis is PC3. Figure 3A represented the condition in positive ion mode and figure 3B for negative ion mode.

A B


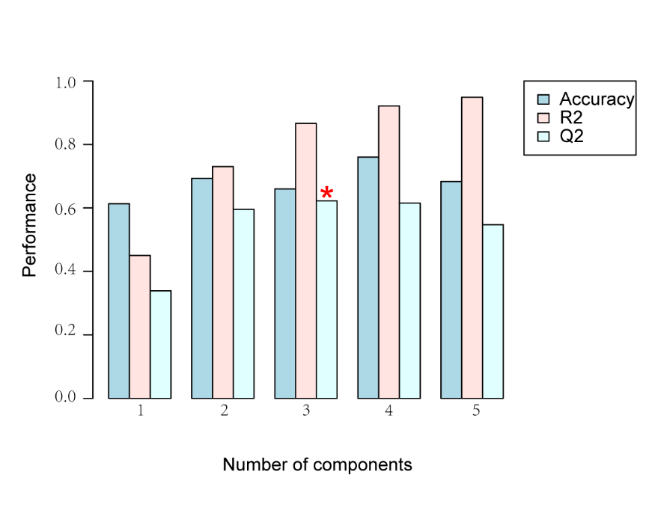

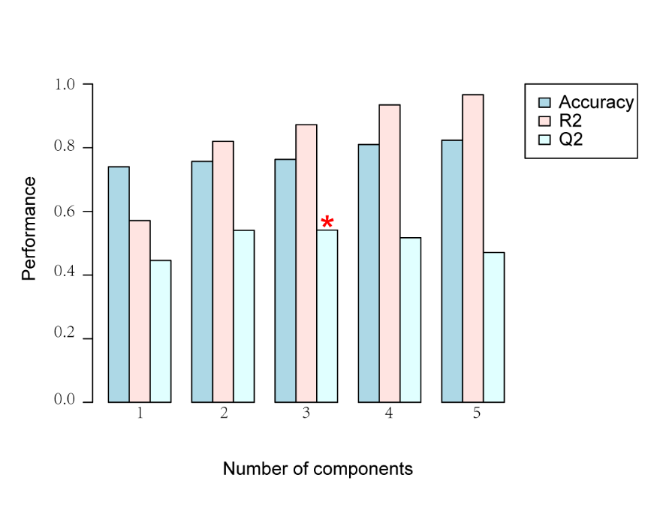


## Supplement figure 4 Evaluation of the PLSDA model among DCM patients, ICM patients and HC.

R2Y and Q2 scores generated in PLS-DA suggested a good stability and predictability of these two models. The asterisk indicated the optimal number of components for modeling. Figure 4A represented the condition in positive ion mode and figure 4B for negative ion mode.

A B

##
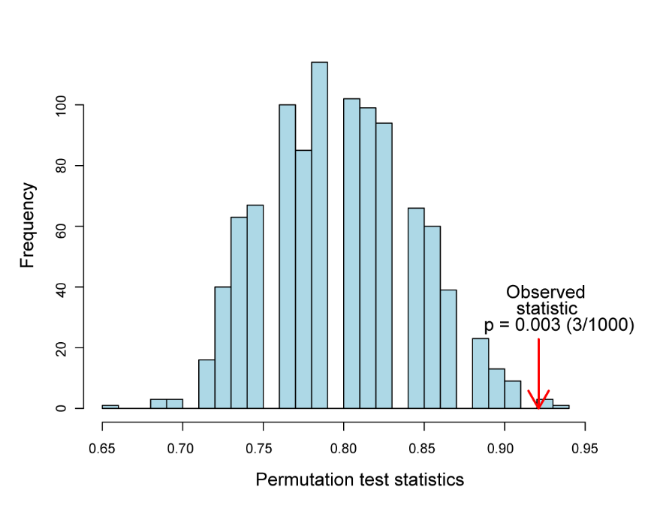

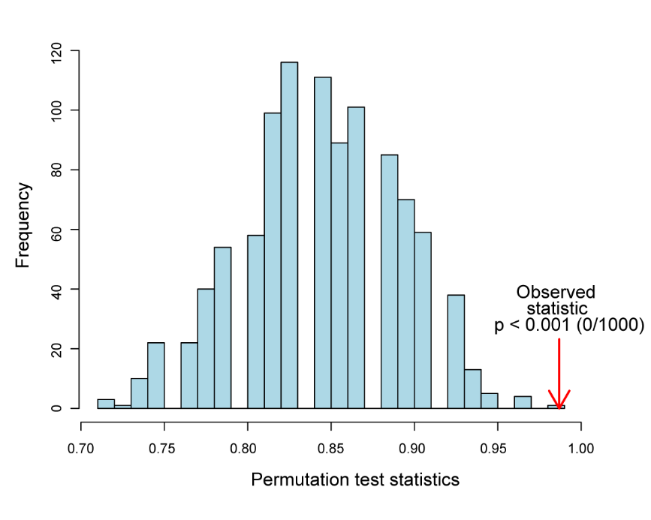


## Supplement figure 5 Permutations plot for PLS-DA models.

## PLS-DA model validation by permutation tests based on prediction accuracy. Figure 5A represented the condition in positive ion mode and figure 5B for negative ion mode.


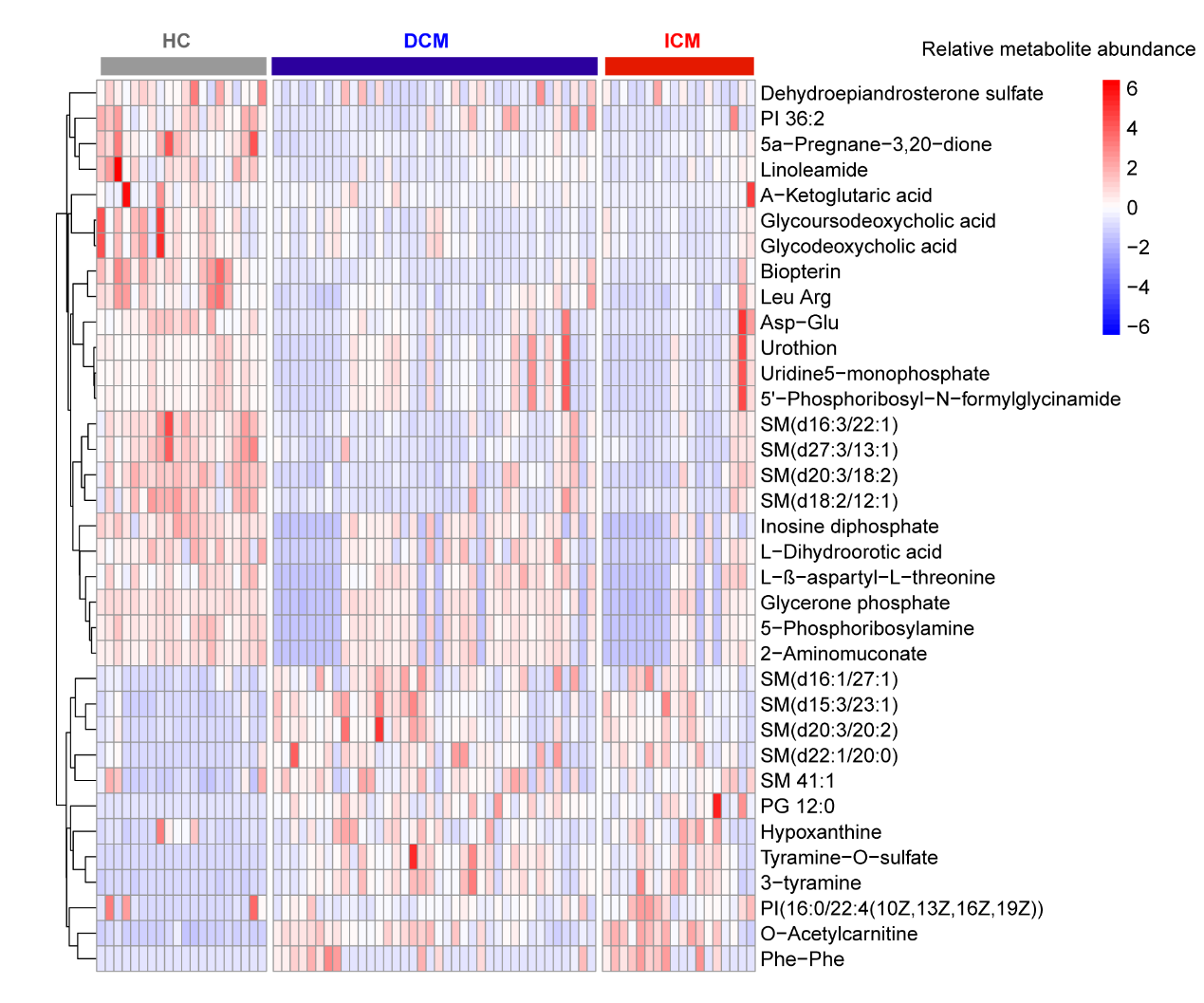


## Supplement figure 6 Heart failure related differential metabolites shared with DCM and ICM patients.

## The heatmap is composed of differential metabolites (FC >2 or <0.5, adjusted P<0.05) shared with DCM and ICM. Each column represents an individual sample from each group. The color intensity is proportional to the original concentration of each metabolites.

# Supplementary Tables

## Supplement table 1 Metabolites with significant changes shared with DCM and ICM.

## Fold change is calculated as the ratio of mean concentration of DCM or ICM relative to that of healthy control group. P value calculated in linear model adjusted for age gender, medication use by Benjamini–Hochberg method.

| English Name | Log2(FC)  DCM/HC | adj.*P*  DCM vs HC | Log2(FC)  ICM/HC | adj.*P*  ICM vs HC |
| --- | --- | --- | --- | --- |
| Tyramine-O-sulfate | 3.7470 | 0.0000 | 3.5182 | 0.0000 |
| 3-tyramine | 2.9693 | 0.0000 | 2.5968 | 0.0000 |
| PG 12:0 | 3.8494 | 0.0000 | 3.6260 | 0.0000 |
| Biopterin | -1.9722 | 0.0000 | -1.9278 | 0.0000 |
| O-Acetylcarnitine | 2.1016 | 0.0000 | 2.6500 | 0.0000 |
| SM(d16:3/22:1) | -1.6016 | 0.0000 | -1.4216 | 0.0005 |
| SM(d15:3/23:1) | 1.1178 | 0.0000 | 1.1764 | 0.0000 |
| 5α-Pregnane-3,20-dione | -1.4819 | 0.0000 | -1.2802 | 0.0005 |
| SM(d27:3/13:1) | -2.1399 | 0.0000 | -2.3113 | 0.0001 |
| Phenethylamine | 0.8766 | 0.0000 | 1.0038 | 0.0003 |
| SM(d20:3/20:2) | 1.2425 | 0.0000 | 1.3362 | 0.0000 |
| SM d38:3 | 0.7459 | 0.0000 | 0.7955 | 0.0008 |
| 2-hydroxybutyric acid | 0.7751 | 0.0000 | 1.1168 | 0.0001 |
| 11β-Hydroxyprogesterone | 0.7744 | 0.0000 | 0.8424 | 0.0006 |
| Myristoylglycine | 0.4510 | 0.0000 | 0.5645 | 0.0001 |
| lysoPC 18:2 | -0.5563 | 0.0000 | -0.5813 | 0.0005 |
| SM(d20:3/18:2) | -1.7860 | 0.0000 | -2.1010 | 0.0025 |
| DG 28:5 | -0.5521 | 0.0000 | -0.5933 | 0.0025 |
| Acetylcholine | -0.7362 | 0.0000 | -0.4625 | 0.0286 |
| SM(d18:2/12:1) | -2.0749 | 0.0000 | -1.8383 | 0.0071 |
| Asp-Glu | -2.2462 | 0.0000 | -2.3217 | 0.0013 |
| 4-Oxoretinol | 0.5979 | 0.0000 | 0.4250 | 0.0040 |
| SM d40:4 | 0.6471 | 0.0001 | 0.6447 | 0.0025 |
| (9Z)-(7S,8S)-Dihydroxyoctadecenoic acid | -0.7195 | 0.0001 | -0.7211 | 0.0135 |
| LysoPC(18:0) | -0.5337 | 0.0001 | -0.5583 | 0.0015 |
| Sulfate | -0.5734 | 0.0001 | -0.5407 | 0.0103 |
| Glycoursodeoxycholic acid | -1.4855 | 0.0001 | -1.3059 | 0.0068 |
| Biliverdin | 0.6491 | 0.0001 | 0.8772 | 0.0025 |
| SM(d21:0/20:1) | 0.8002 | 0.0002 | 1.0506 | 0.0008 |
| lysoPC 20:3 | -0.5658 | 0.0003 | -0.6934 | 0.0015 |
| lysoPC 22:5 | -0.7278 | 0.0003 | -0.9764 | 0.0003 |
| SM(d22:1/20:0) | 1.6964 | 0.0003 | 1.9617 | 0.0008 |
| 3-Dehydroquinate | 0.8916 | 0.0004 | 0.9149 | 0.0173 |
| 4-tert-Octylphenol | 0.3890 | 0.0004 | 0.4090 | 0.0040 |
| Leu Arg | -1.1553 | 0.0004 | -1.1864 | 0.0028 |
| Linoleamide | -1.3744 | 0.0004 | -1.4171 | 0.0171 |
| Isodesmosine | 0.9966 | 0.0005 | 0.8473 | 0.0094 |
| Dl-Citrulline | -0.7430 | 0.0006 | -0.6618 | 0.0029 |
| Biliverdin | 0.6636 | 0.0006 | 0.7964 | 0.0074 |
| lysoPC 20:4 | -0.5518 | 0.0006 | -0.4433 | 0.0357 |
| SM d43:2 | 1.2823 | 0.0008 | 1.3746 | 0.0055 |
| 9(10)-EpOME | -0.4967 | 0.0009 | -0.5605 | 0.0134 |
| PI(16:0/22:4(10Z,13Z,16Z,19Z)) | 1.6868 | 0.0010 | 2.4060 | 0.0070 |
| plasmenyl-PC 32:1 | 0.5056 | 0.0010 | 0.7338 | 0.0001 |
| plasmenyl-PC 34:2 | -0.4293 | 0.0010 | -0.3898 | 0.0128 |
| 2E,6Z,8Z,12Z-hexadecatetraenoic acid | -0.4327 | 0.0010 | -0.5503 | 0.0025 |
| 17-Hydroxylinolenic acid | -0.5801 | 0.0012 | -0.6253 | 0.0235 |
| SM 41:1 | 1.0041 | 0.0013 | 1.1818 | 0.0100 |
| 1-Stearoyl-Sn-Glycerol-3-Phosphocholine | -0.4488 | 0.0013 | -0.5056 | 0.0025 |
| SM(d16:2/12:1) | -0.9570 | 0.0013 | -0.8966 | 0.0357 |
| Calcifediol | 0.8363 | 0.0013 | 1.0252 | 0.0049 |
| Estrone glucuronide | 0.8301 | 0.0016 | 0.8580 | 0.0021 |
| N,N-Dimethylglycine | 0.2537 | 0.0016 | 0.4183 | 0.0003 |
| FAHFA 27:0 | -0.9416 | 0.0016 | -1.0800 | 0.0072 |
| 11-Oxooctadecanoic acid | -0.7876 | 0.0016 | -0.9579 | 0.0040 |
| SM(d14:2/14:1) | -0.6576 | 0.0017 | -0.7361 | 0.0265 |
| PC(O-12:0/O-12:0) | -0.7699 | 0.0017 | -0.7282 | 0.0437 |
| D-Pyrrolidine-2-carboxylic acid | -0.8063 | 0.0022 | -0.7975 | 0.0394 |
| Cortolone-3-glucuronide | 0.7066 | 0.0022 | 0.6552 | 0.0141 |
| SM d43:3 | 0.6261 | 0.0023 | 0.8172 | 0.0046 |
| delta-Tocopherol | -0.5070 | 0.0023 | -0.4980 | 0.0171 |
| Glycodeoxycholic acid | -1.1560 | 0.0026 | -1.0043 | 0.0212 |
| DG 36:7 | -0.8676 | 0.0027 | -0.8352 | 0.0239 |
| LPC 19:0 | -0.5748 | 0.0036 | -0.9763 | 0.0050 |
| O-methoxycatechol-O-sulphate | -0.9012 | 0.0036 | -1.4293 | 0.0021 |
| D-Galactose | 0.6085 | 0.0037 | 0.9190 | 0.0021 |
| SM(d29:1/12:0) | 0.7840 | 0.0038 | 0.9633 | 0.0016 |
| 9,10-DHOME | -0.5329 | 0.0039 | -0.6557 | 0.0374 |
| Hypogeic acid | 0.7231 | 0.0042 | 0.9644 | 0.0080 |
| PA(O-18:0/19:0) | 0.6579 | 0.0042 | 0.7629 | 0.0213 |
| SM(d20:0/22:2) | 0.4709 | 0.0048 | 0.5090 | 0.0095 |
| Glycerone phosphate | -1.1195 | 0.0052 | -1.7603 | 0.0032 |
| Hypoxanthine | 1.0045 | 0.0057 | 1.4157 | 0.0080 |
| 1-Palmitoyl-Sn-Glycero-3-Phosphocholine | -0.3940 | 0.0060 | -0.3902 | 0.0226 |
| Alloxan | -0.9121 | 0.0067 | -1.1901 | 0.0057 |
| Dl-Cystine | 0.6296 | 0.0069 | 0.7567 | 0.0308 |
| Prolylglycine | 0.5572 | 0.0076 | 0.8949 | 0.0040 |
| FAHFA 24:0 | -0.5403 | 0.0077 | -0.9339 | 0.0040 |
| PI 36:2 | -1.1049 | 0.0077 | -1.8739 | 0.0004 |
| 2-hydroxyhexadecanoic acid | -0.3626 | 0.0083 | -0.4125 | 0.0134 |
| Uridine5-monophosphate | -1.0263 | 0.0090 | -1.5275 | 0.0057 |
| Isocitric acid | 0.9145 | 0.0094 | 1.1378 | 0.0130 |
| 15-Methylpalmitic Acid | 0.4510 | 0.0094 | 0.5294 | 0.0091 |
| Glycerophosphoric acid | -0.3020 | 0.0094 | -0.3173 | 0.0173 |
| 2,3-Dihydroxypropyl 12-methyltridecanoate | 0.4127 | 0.0095 | 0.5802 | 0.0043 |
| Phe-Phe | 2.1406 | 0.0095 | 3.3135 | 0.0040 |
| Progesterone | 0.4122 | 0.0103 | 0.5625 | 0.0041 |
| SM(d18:0/18:1(9Z)) | 0.3468 | 0.0113 | 0.3883 | 0.0487 |
| 8(R)-HPODE | -0.5335 | 0.0114 | -0.6732 | 0.0241 |
| Urothion | -1.2933 | 0.0114 | -1.8153 | 0.0057 |
| PC 17:0e | -0.3800 | 0.0114 | -0.3878 | 0.0365 |
| PI(16:0/22:4(10Z,13Z,16Z,19Z)) | -1.9840 | 0.0117 | -2.9283 | 0.0075 |
| 4-phenylbutanic acid-O-sulphate | -0.7835 | 0.0124 | -1.1790 | 0.0103 |
| A-Ketoglutaric acid oxime | -1.4487 | 0.0125 | -2.3433 | 0.0048 |
| 14-Methylhexadecanoic Acid | 0.5088 | 0.0129 | 0.6022 | 0.0129 |
| Palmitic acid | 0.3601 | 0.0129 | 0.5375 | 0.0053 |
| L-Erythrulose | 0.3495 | 0.0129 | 0.5452 | 0.0091 |
| Dehydroepiandrosterone sulfate | -1.0083 | 0.0130 | -1.0898 | 0.0234 |
| lysoPC 16:1 | -0.4585 | 0.0133 | -0.6792 | 0.0046 |
| Creatinine | 0.2939 | 0.0133 | 0.3849 | 0.0212 |
| Glycoursodeoxycholic acid | -1.1661 | 0.0138 | -1.1236 | 0.0260 |
| Oleoyl-L-α-lysophosphatidic acid | 0.5411 | 0.0155 | 0.6795 | 0.0354 |
| 5'-Phosphoribosyl-N-formylglycinamide | -1.5373 | 0.0155 | -2.0712 | 0.0081 |
| IDP | -1.9132 | 0.0155 | -2.9512 | 0.0021 |
| LPG 9:0 | 0.6475 | 0.0161 | 0.8498 | 0.0116 |
| trans-9-Octadecenoic acid | 0.5616 | 0.0161 | 0.9387 | 0.0040 |
| D-Phenylalanine | 0.2402 | 0.0169 | 0.4045 | 0.0040 |
| PA 36:2 | 0.6568 | 0.0195 | 0.7166 | 0.0473 |
| Cis-11,14-Eicosadienoic acid | 0.4818 | 0.0195 | 0.7250 | 0.0129 |
| Melanin | -0.9388 | 0.0211 | -1.1301 | 0.0031 |
| FAHFA 25:0 | -0.5225 | 0.0216 | -0.7002 | 0.0345 |
| L-β-aspartyl-L-threonine | -2.4154 | 0.0230 | -3.5534 | 0.0073 |
| SM d37:1 | 0.3051 | 0.0233 | 0.4262 | 0.0420 |
| Stearidonic acid | -0.3409 | 0.0253 | -0.5703 | 0.0068 |
| Dl-P-Hydroxyphenyl lactic acid | 0.3522 | 0.0255 | 0.5087 | 0.0087 |
| Cis-10-Nonadecenoic acid | 0.5436 | 0.0266 | 0.7121 | 0.0249 |
| trans,trans-Muconic acid | 0.3297 | 0.0273 | 0.5231 | 0.0094 |
| 3α,7α,12α,26-Tetrahydroxy-5β-cholestane | -0.6265 | 0.0273 | -0.6335 | 0.0206 |
| Methylarsonate | -0.6192 | 0.0289 | -0.8238 | 0.0188 |
| Bilirubin | 0.7641 | 0.0299 | 1.2466 | 0.0058 |
| 2-Aminomuconate | -2.4646 | 0.0303 | -4.0173 | 0.0056 |
| (11E)-Octadecenoic acid | 0.4942 | 0.0315 | 0.3986 | 0.0398 |
| Pseudouridine | 0.3324 | 0.0330 | 0.4556 | 0.0302 |
| Quinolinic acid | -0.8899 | 0.0330 | -1.7102 | 0.0040 |
| LysoPC(15:0) | -0.3315 | 0.0347 | -0.5173 | 0.0068 |
| L-Dihydroorotic acid | -1.1621 | 0.0351 | -1.6943 | 0.0116 |
| 9-Oxoode | -0.3889 | 0.0358 | -0.5020 | 0.0173 |
| Prostaglandin E1 | -0.8327 | 0.0364 | -1.0661 | 0.0292 |
| PC 37:0e | 0.3168 | 0.0386 | 0.4876 | 0.0162 |
| plasmenyl-PC 36:1 | 0.1902 | 0.0407 | 0.2349 | 0.0439 |
| Ursodeoxycholate | -0.9491 | 0.0419 | -1.8929 | 0.0138 |
| Lactosylceramide(18:1/16:0) | 0.1950 | 0.0422 | 0.3011 | 0.0156 |
| Dl-Glyceraldehyde3-phosphate | 0.7642 | 0.0434 | 1.3998 | 0.0074 |
| 1-Oleoylglycerophosphoinositol | -0.3809 | 0.0447 | -0.5484 | 0.0058 |
| 5-Phosphoribosylamine | -1.1552 | 0.0447 | -1.7889 | 0.0053 |
| Phosphatidyl-N-dimethylethanolamine | -0.2159 | 0.0452 | -0.3043 | 0.0437 |
| N6-Acetyl-L-lysine | 0.1633 | 0.0460 | 0.3016 | 0.0234 |
| 2-Ketobutyric acid | 0.2760 | 0.0468 | 0.4132 | 0.0380 |

## Supplement table 2 DCM-specific metabolites in positive and negative ion mode.

## Fold change is calculated as the ratio of mean concentration of DCM/ICM relative to that of HC group. *P* value calculated in linear model adjusted for age gender, medication use by Benjamini–Hochberg method.

| English Name | Log2FC  DCM/HC | adj.*P* DCM/HC | Log2FC  ICM/HC | adj.*P*  ICM/HC |
| --- | --- | --- | --- | --- |
| PC 37:5e | 1.23 | 0.0001 | 0.43 | 0.4137 |
| Cis-5-Dodecenoic acid | -0.44 | 0.0002 | -0.22 | 0.2244 |
| Mono-ethylhexylphthalate | -0.81 | 0.0004 | -0.70 | 0.0534 |
| SM d28:2 | -0.98 | 0.0005 | -0.85 | 0.0533 |
| PC 33:2 | -0.68 | 0.0006 | -0.45 | 0.2325 |
| Linoleoyl ethanolamide | -0.45 | 0.0009 | -0.23 | 0.1886 |
| 1-Pyrroline-2-carboxylate | -0.47 | 0.0013 | -0.21 | 0.3994 |
| Oxalosuccinate | 0.43 | 0.0015 | 0.36 | 0.0720 |
| PE (16:0/18:1(9Z)) | 0.65 | 0.0017 | 0.14 | 0.7690 |
| Nε,Nε,Nε-trimethyllysine | -0.49 | 0.0020 | -0.39 | 0.1469 |
| 8-oxo-octadecanoic acid | -0.66 | 0.0022 | -0.58 | 0.0758 |
| SM d41:3 | 0.33 | 0.0022 | 0.34 | 0.0910 |
| cis-5, cis-12-octadecadienoic acid | -0.54 | 0.0023 | -0.51 | 0.0713 |
| Glycine | -0.35 | 0.0023 | -0.27 | 0.0758 |
| Vernolic acid | -0.43 | 0.0023 | -0.21 | 0.4074 |
| Phosphatidylethanolamine (20:1/16:1) | -0.32 | 0.0024 | -0.17 | 0.3285 |
| SM 38:0 | 0.30 | 0.0027 | 0.33 | 0.0758 |
| 25-hydroxy-cholesterol(d3) | -0.90 | 0.0030 | -0.94 | 0.0632 |
| α-Linolenic acid | -0.69 | 0.0030 | -0.48 | 0.1761 |
| D-Ornithine | -0.41 | 0.0036 | -0.31 | 0.1042 |
| 4-Vinylphenol sulfate | -1.01 | 0.0036 | -0.63 | 0.1212 |
| PC 33:1 | 0.36 | 0.0038 | 0.14 | 0.5052 |
| PC 33:0 | 0.60 | 0.0052 | 0.43 | 0.2033 |
| Deoxycholic acid | -1.04 | 0.0060 | -0.83 | 0.1981 |
| SM d29:3 | -0.76 | 0.0060 | -0.48 | 0.2626 |
| Cer 42:2 | 1.84 | 0.0076 | 1.83 | 0.1012 |
| D(-)-Arginine | -0.33 | 0.0077 | -0.23 | 0.1693 |
| MG 20:1 | 1.38 | 0.0081 | 1.59 | 0.1079 |
| plasmenyl-PC 33:0 | 0.30 | 0.0081 | 0.17 | 0.3110 |
| Pyrrole-2-carboxylic acid | -0.38 | 0.0089 | -0.38 | 0.0561 |
| D-Norvaline | 0.27 | 0.0089 | 0.17 | 0.1826 |
| 13-HODE | -0.58 | 0.0089 | -0.52 | 0.0689 |
| 10-Oxooctadecanoic acid | -0.28 | 0.0102 | -0.25 | 0.0816 |
| DG 36:7 | -0.81 | 0.0104 | -0.58 | 0.1866 |
| SM d28:3 | -0.76 | 0.0113 | -0.68 | 0.1079 |
| (±)-3-Methyl-2-oxovaleric acid | 0.34 | 0.0122 | 0.34 | 0.0699 |
| FAHFA 34:4 | -0.46 | 0.0129 | -0.43 | 0.0646 |
| DG (14:1n5/0:0/18:2n6) | -0.55 | 0.0129 | -0.35 | 0.2419 |
| 3β-Hydroxy-D5-Cholenic acid | -0.64 | 0.0129 | -0.53 | 0.0564 |
| PC (16:0/22:5(4Z,7Z,10Z,13Z,16Z)) | -0.36 | 0.0131 | -0.34 | 0.0833 |
| SM d35:0 | 0.48 | 0.0149 | 0.36 | 0.2343 |
| PC 35:4 | -0.63 | 0.0151 | 0.19 | 0.5740 |
| 21-Deoxycortisol | 1.20 | 0.0155 | 0.62 | 0.3591 |
| plasmenyl-PC 35:1 | 0.28 | 0.0163 | 0.28 | 0.1469 |
| PG 16:0; PG 3:0-13:0 | 0.21 | 0.0168 | 0.24 | 0.0843 |
| PC 39:6 | 0.35 | 0.0183 | 0.27 | 0.1987 |
| Cer 42:1 | 1.12 | 0.0185 | 1.03 | 0.1214 |
| FAHFA 26:1 | -0.57 | 0.0195 | -0.57 | 0.0646 |
| LysoPE(0:0/20:5(5Z,8Z,11Z,14Z,17Z)) | -0.46 | 0.0197 | -0.33 | 0.2325 |
| Glycocholic acid | -1.04 | 0.0238 | -0.90 | 0.1693 |
| SM d31:3 | -0.68 | 0.0248 | -0.68 | 0.1608 |
| Phenylacetyl-L-glutamine | 0.66 | 0.0248 | 0.55 | 0.2193 |
| 10Z,12E-tetradecadienoic acid | -0.40 | 0.0249 | -0.25 | 0.3893 |
| 1-Pyrroline | -0.33 | 0.0249 | -0.25 | 0.1608 |
| 3-Methoxy-4-Hydroxyphenylethyleneglycol Sulfate | 0.54 | 0.0255 | 0.52 | 0.0618 |
| FAHFA 27:0 | -0.58 | 0.0255 | -0.49 | 0.0728 |
| PC 32:1 | 0.37 | 0.0260 | 0.10 | 0.7847 |
| PC 34:2 | -0.22 | 0.0260 | -0.19 | 0.1971 |
| plasmenyl-PC 32:0 | 0.24 | 0.0265 | 0.18 | 0.3003 |
| Tetrahydroaldosterone-3-glucuronide | 0.32 | 0.0273 | 0.05 | 0.8126 |
| (R)-2-Hydroxyisocaproate | -0.73 | 0.0273 | -0.50 | 0.0932 |
| 1,4-Bis(2-ethylhexyl) sulfosuccinate | 1.08 | 0.0273 | 0.81 | 0.1770 |
| PC 36:5 | -0.29 | 0.0277 | -0.29 | 0.1183 |
| L-Aspartate | -0.62 | 0.0300 | -0.60 | 0.1608 |
| PC 36:4 | -0.25 | 0.0300 | -0.29 | 0.1469 |
| PC 37:5 | 0.37 | 0.0300 | -0.02 | 0.9481 |
| L-Palmitoylcarnitine | 0.29 | 0.0336 | 0.32 | 0.1327 |
| 2'-Deoxyuridine | -0.23 | 0.0336 | -0.15 | 0.3833 |
| PC (14:1(9Z)/22:2(13Z,16Z)) | -0.15 | 0.0340 | -0.08 | 0.5305 |
| plasmenyl-PC 38:5 | 0.23 | 0.0340 | 0.06 | 0.7690 |
| SM 40:0 | 0.53 | 0.0343 | 0.43 | 0.1534 |
| plasmenyl-PC 40:1 | -0.51 | 0.0368 | -0.64 | 0.0758 |
| FAHFA 40:3 | 0.28 | 0.0373 | 0.28 | 0.0714 |
| N-Palmitoyl phenylalanine | 0.52 | 0.0386 | 0.34 | 0.4370 |
| PE 36:4 | 0.84 | 0.0403 | 0.92 | 0.1118 |
| PC 36:2 | -0.17 | 0.0405 | -0.13 | 0.3261 |
| PC 32:0e | 0.20 | 0.0405 | 0.26 | 0.0632 |
| PC 35:1 | 0.47 | 0.0405 | 0.46 | 0.2510 |
| plasmenyl-PC 40:5 | 0.22 | 0.0405 | 0.16 | 0.2967 |
| Dodecanedioic acid | -0.23 | 0.0407 | -0.14 | 0.3285 |
| SM d39:1 | 0.30 | 0.0409 | 0.31 | 0.1079 |
| (Rs)-Mevalonic acid | -0.31 | 0.0410 | -0.13 | 0.5553 |
| Ethyl caprate | 0.37 | 0.0419 | 0.34 | 0.1172 |
| Nα-Methylhistidine | -0.28 | 0.0421 | -0.15 | 0.3650 |
| TAG 39:9 | 0.81 | 0.0434 | 0.81 | 0.2064 |
| FAHFA 32:4 | -0.37 | 0.0434 | -0.28 | 0.0832 |
| L-Asparagine | 0.78 | 0.0434 | 0.83 | 0.1563 |
| Hyocholate | -0.55 | 0.0447 | -0.09 | 0.7367 |
| (±)-Myristoylcarnitine | 0.47 | 0.0452 | 0.16 | 0.6933 |
| Phosphatidylinositol 18:0-20:3 | 1.15 | 0.0460 | 0.76 | 0.3616 |
| SM d40:5 | -0.52 | 0.0462 | -0.42 | 0.2626 |

## Supplement table 3 ICM-specific metabolites in positive and negative ion mode.

## Fold change is calculated as the ratio of mean concentration of ICM/DCM relative to that of HC group. *P* value calculated in linear model adjusted for age by Benjamini–Hochberg method.

| English Name | Log2FC  DCM/HC | adj.*P* DCM/HC | Log2FC  ICM/HC | adj.*P*  ICM/HC |
| --- | --- | --- | --- | --- |
| PG(6:0/8:0) | 0.26 | 0.0974 | 0.87 | 0.0001 |
| PC 38:5 | 0.16 | 0.1102 | 0.43 | 0.0034 |
| L-Glutamic acid | 0.39 | 0.1067 | 0.93 | 0.0040 |
| α-D-Galactose 1-phosphate | 1.19 | 0.0734 | 2.71 | 0.0040 |
| 3-Guanidinopropanoate | -0.41 | 0.0984 | -0.80 | 0.0046 |
| 1-Palmitoylglycerophosphoinositol | -0.24 | 0.1801 | -0.51 | 0.0056 |
| N-Acetylneuraminic acid | 0.19 | 0.1431 | 0.48 | 0.0056 |
| β-Linoleic acid | 0.32 | 0.1017 | 0.68 | 0.0071 |
| L-Phenylalanine | 0.19 | 0.0653 | 0.35 | 0.0091 |
| PA 37:10 | 0.25 | 0.0974 | 0.46 | 0.0091 |
| Prostaglandin A2-d4 | 0.13 | 0.7332 | 0.62 | 0.0120 |
| PI 36:4 | -0.61 | 0.1242 | -1.44 | 0.0126 |
| plasmenyl-PE 36:4 | -0.60 | 0.1710 | -1.44 | 0.0129 |
| Oxypurinol | 0.53 | 0.1288 | 0.88 | 0.0129 |
| FAHFA 24:1 | 0.09 | 0.2093 | 0.28 | 0.0133 |
| PE 38:5e | -0.98 | 0.0992 | -2.39 | 0.0140 |
| N7-Methylguanosine | 0.08 | 0.5461 | 0.46 | 0.0143 |
| 11β-Prostaglandin F2α | 0.32 | 0.2093 | 0.77 | 0.0145 |
| PE 21:5 | 0.17 | 0.3363 | 0.67 | 0.0159 |
| LPI 18:2 | -0.28 | 0.0970 | -0.49 | 0.0173 |
| N-Acetyl-L-glutamic acid | 0.24 | 0.2136 | 0.56 | 0.0173 |
| PC 38:4 | 0.10 | 0.3074 | 0.30 | 0.0173 |
| Arachidonic acid | 0.22 | 0.0639 | 0.43 | 0.0188 |
| Phosphatidylcholine 18:0-18:3 | -0.02 | 0.9555 | 0.83 | 0.0188 |
| PI 34:2 | -0.58 | 0.1433 | -1.66 | 0.0188 |
| PE(20:4(5Z,8Z,11Z,14Z)/P-18:1(11Z)) | -0.74 | 0.1288 | -1.68 | 0.0206 |
| PG 15:0 | -0.04 | 0.8594 | -0.49 | 0.0211 |
| trans-Vaccenic acid | 0.98 | 0.1036 | 1.69 | 0.0212 |
| SM 34:2 | -0.09 | 0.2790 | -0.24 | 0.0213 |
| Citric acid | -0.77 | 0.0689 | -1.14 | 0.0218 |
| PC(14:0/20:5(5Z,8Z,11Z,14Z,17Z)) | -0.03 | 0.9616 | -0.85 | 0.0226 |
| LPA 23:0 | -0.34 | 0.4957 | -1.37 | 0.0226 |
| D-Glucuronate 1-phosphate | 0.17 | 0.3674 | 0.48 | 0.0229 |
| Ascorbic acid-2-sulfate | 0.35 | 0.0616 | 0.57 | 0.0234 |
| FAHFA 41:4 | 0.66 | 0.1564 | 1.25 | 0.0234 |
| PA 32:0 | 0.34 | 0.0609 | 0.55 | 0.0234 |
| N-Methyl-L-glutamic acid | 0.33 | 0.0621 | 0.65 | 0.0234 |
| plasmenyl-PC 38:1 | 0.59 | 0.0997 | 1.01 | 0.0242 |
| Pyroglutamic acid | 0.04 | 0.6924 | 0.23 | 0.0247 |
| PE 44:1; PE 27:0-17:1 | -0.85 | 0.0526 | -1.16 | 0.0249 |
| CerP 34:2 | -0.34 | 0.2665 | -1.18 | 0.0250 |
| FAHFA 36:3 | 0.57 | 0.2188 | 1.22 | 0.0270 |
| FA 19:2 | 0.31 | 0.1433 | 0.60 | 0.0280 |
| plasmenyl-PC 38:0 | 0.71 | 0.0604 | 1.07 | 0.0299 |
| Sphingosine-1-phosphate | -0.34 | 0.0754 | -0.63 | 0.0299 |
| Hexacosanoic acid | -0.68 | 0.2595 | -1.52 | 0.0305 |
| D-Pyrrolidine-2-carboxylic acid | 0.28 | 0.0734 | 0.37 | 0.0306 |
| L-Homoserine | -0.05 | 0.6406 | 0.27 | 0.0331 |
| PC 36:6 | -0.09 | 0.6024 | -0.55 | 0.0357 |
| FAHFA 33:4 | -0.48 | 0.0532 | -0.65 | 0.0364 |
| LPA 20:5 | -0.40 | 0.2939 | -0.88 | 0.0370 |
| L-Glutamine | 0.05 | 0.6514 | 0.29 | 0.0370 |
| PC 37:4 | 0.23 | 0.0952 | 0.42 | 0.0394 |
| Tetrahydro-11-deoxycortisol | -0.30 | 0.4336 | -1.19 | 0.0396 |
| SM d40:1 | 0.28 | 0.0561 | 0.36 | 0.0397 |
| Dl-2-Amino-3-phosphonopropionic acid | -0.23 | 0.3511 | -0.62 | 0.0403 |
| 5(S)-Hpete | -0.21 | 0.3995 | -0.95 | 0.0403 |
| PA 20:0 | 0.14 | 0.0578 | 0.22 | 0.0425 |
| plasmenyl-PE 34:2 | 1.06 | 0.0865 | 1.42 | 0.0425 |
| FAHFA 38:4; FAHFA 21:2/17:2 | 0.77 | 0.1865 | 1.46 | 0.0437 |
| FAHFA 40:5 | 0.24 | 0.6119 | 0.92 | 0.0450 |
| LPA 18:2 | 0.20 | 0.1242 | 0.33 | 0.0455 |
| 5α-Cholestan-3-one | 0.09 | 0.5962 | 0.35 | 0.0460 |
| plasmenyl-PC 42:6 | 0.86 | 0.0788 | 1.32 | 0.0491 |

## Supplement table 4. Related pathway observed in DCM.

| Pathway | *P* value | Impact |
| --- | --- | --- |
| Linoleic acid metabolism | 0.0019 | 0.00 |
| Glycerophospholipid metabolism | 0.0042 | 0.32 |
| D-Arginine and D-ornithine metabolism | 0.0193 | 0.00 |
| alpha-Linolenic acid metabolism | 0.0375 | 0.33 |
| Arginine biosynthesis | 0.2000 | 0.23 |
| Nicotinate and nicotinamide metabolism | 0.2223 | 0.00 |
| Alanine, aspartate and glutamate metabolism | 0.2289 | 0.22 |
| Glycerolipid metabolism | 0.2448 | 0.04 |
| Porphyrin and chlorophyll metabolism | 0.2615 | 0.13 |
| Primary bile acid biosynthesis | 0.2895 | 0.06 |
| Glycine, serine and threonine metabolism | 0.3114 | 0.32 |
| Citrate cycle (TCA cycle) | 0.3350 | 0.04 |
| Purine metabolism | 0.3416 | 0.10 |
| Sphingolipid metabolism | 0.3572 | 0.00 |
| Sulfur metabolism | 0.3878 | 0.21 |
| Valine, leucine and isoleucine biosynthesis | 0.3878 | 0.00 |
| Steroid hormone biosynthesis | 0.3920 | 0.13 |
| Propanoate metabolism | 0.4008 | 0.04 |
| Pyrimidine metabolism | 0.4113 | 0.18 |
| Phenylalanine metabolism | 0.4587 | 0.24 |
| Aminoacyl-tRNA biosynthesis | 0.5513 | 0.00 |
| Glyoxylate and dicarboxylate metabolism | 0.5765 | 0.11 |
| Glycosylphosphatidylinositol (GPI)-anchor biosynthesis | 0.5770 | 0.00 |
| Histidine metabolism | 0.6262 | 0.00 |
| Biosynthesis of unsaturated fatty acids | 0.6418 | 0.00 |
| Terpenoid backbone biosynthesis | 0.6697 | 0.11 |
| Arginine and proline metabolism | 0.6714 | 0.00 |
| Fatty acid degradation | 0.6855 | 0.00 |
| Pantothenate and CoA biosynthesis | 0.6895 | 0.00 |
| Fructose and mannose metabolism | 0.7082 | 0.00 |
| beta-Alanine metabolism | 0.7257 | 0.00 |
| Lysine degradation | 0.7861 | 0.00 |
| Glycolysis / Gluconeogenesis | 0.7990 | 0.00 |
| Galactose metabolism | 0.8111 | 0.05 |
| Glutathione metabolism | 0.8225 | 0.09 |
| Inositol phosphate metabolism | 0.8433 | 0.00 |
| Cysteine and methionine metabolism | 0.8701 | 0.06 |
| Arachidonic acid metabolism | 0.8924 | 0.00 |
| Amino sugar and nucleotide sugar metabolism | 0.8989 | 0.00 |
| Fatty acid elongation | 0.9108 | 0.00 |
| Tryptophan metabolism | 0.9214 | 0.00 |
| Steroid biosynthesis | 0.9262 | 0.00 |
| Fatty acid biosynthesis | 0.9461 | 0.01 |

## Supplement table 5. Related pathway observed in ICM.

| Pathway | *P* value | Impact |
| --- | --- | --- |
| Linoleic acid metabolism | 0.0015 | 1.00 |
| Glycerophospholipid metabolism | 0.0026 | 0.32 |
| Arginine biosynthesis | 0.0055 | 0.35 |
| Nitrogen metabolism | 0.0386 | 0.00 |
| D-Glutamine and D-glutamate metabolism | 0.0386 | 0.50 |
| Alanine, aspartate and glutamate metabolism | 0.0630 | 0.31 |
| Glyoxylate and dicarboxylate metabolism | 0.0939 | 0.03 |
| Phenylalanine metabolism | 0.1005 | 0.60 |
| Sphingolipid metabolism | 0.1038 | 0.02 |
| Purine metabolism | 0.1411 | 0.10 |
| alpha-Linolenic acid metabolism | 0.1568 | 0.00 |
| Phenylalanine, tyrosine and tryptophan biosynthesis | 0.2021 | 0.50 |
| Glycerolipid metabolism | 0.2175 | 0.04 |
| Porphyrin and chlorophyll metabolism | 0.2246 | 0.13 |
| Citrate cycle (TCA cycle) | 0.3008 | 0.14 |
| Biosynthesis of unsaturated fatty acids | 0.3158 | 0.00 |
| Arachidonic acid metabolism | 0.3158 | 0.42 |
| Pyrimidine metabolism | 0.3621 | 0.13 |
| Propanoate metabolism | 0.3626 | 0.04 |
| Valine, leucine and isoleucine biosynthesis | 0.3638 | 0.00 |
| Sulfur metabolism | 0.3638 | 0.21 |
| Galactose metabolism | 0.4415 | 0.09 |
| Glutathione metabolism | 0.4605 | 0.03 |
| Aminoacyl-tRNA biosynthesis | 0.4964 | 0.00 |
| Steroid hormone biosynthesis | 0.5050 | 0.10 |
| Glycosylphosphatidylinositol (GPI)-anchor biosynthesis | 0.5475 | 0.00 |
| Glycine, serine and threonine metabolism | 0.5493 | 0.07 |
| Butanoate metabolism | 0.5726 | 0.00 |
| Nicotinate and nicotinamide metabolism | 0.5726 | 0.00 |
| Histidine metabolism | 0.5962 | 0.00 |
| Amino sugar and nucleotide sugar metabolism | 0.6128 | 0.02 |
| Arginine and proline metabolism | 0.6275 | 0.09 |
| Fructose and mannose metabolism | 0.6786 | 0.00 |
| Glycolysis / Gluconeogenesis | 0.7721 | 0.00 |
| Inositol phosphate metabolism | 0.8189 | 0.00 |
| Cysteine and methionine metabolism | 0.8476 | 0.06 |
| Fatty acid elongation | 0.8922 | 0.00 |
| Fatty acid degradation | 0.8922 | 0.00 |
| Tryptophan metabolism | 0.9040 | 0.00 |
| Steroid biosynthesis | 0.9094 | 0.00 |
| Primary bile acid biosynthesis | 0.9282 | 0.04 |
| Fatty acid biosynthesis | 0.9323 | 0.01 |
